# Evaluation of a short in-person and online antenatal educational intervention for high-risk pregnant women linked to antenatal consultation

**DOI:** 10.18332/ejm/175927

**Published:** 2024-01-18

**Authors:** Maria A. Heim, Maria Y. Makuch

**Affiliations:** 1 Department of Obstetrics and Gynecology, University of Campinas Faculty of Medical Sciences, Sao Paulo, Brazil; 2 Campinas Reproductive Health Research Center, Sao Paulo, Brazil

**Keywords:** antenatal education, anxiety, breathing exercises, labor pain, upright position

## Abstract

**INTRODUCTION:**

The aim of this study was to evaluate a short educational intervention that focused on labor pain (through visual analogue scale, VAS), postpartum anxiety, and birthing experience before and during the SARS-CoV-2 (COVID-19) pandemic.

**METHODS:**

This was a quasi-experimental study conducted between November 2019 and May 2021 in Brazil in 100 women with a high-risk pregnancy in the third trimester of pregnancy with an intervention group with in-person or virtual sessions (during the COVID-19 pandemic) and a non-intervention group. The antenatal intervention included breathing and relaxation techniques, upright positions, and information about labor. For evaluation, an antenatal questionnaire, State-Trait Anxiety Inventory (STAI) and a postpartum questionnaire were used. For data analysis, Student's t-test, chi-squared and Fisher's exact tests, ANOVA, bivariate, and multivariate regression analysis, were used.

**RESULTS:**

When comparing the women in the intervention group to the non-intervention group, it was observed that the latter group reported higher fear of pain at labor during antenatal consultations (p<0.013); more women needed analgesia at 0–4 cm dilation (17/40) (p<0.018); the duration of labor was ≥12 hours (37/50) (p<0.037); while the intervention reported having a regular, good or excellent labor period (36/50) (p=0.014). The multiple regression analysis for labor pain showed a significant relationship between mode of delivery (cesarean delivery: RR; SE -21.43; 5.32, p<0.001) and labor pain, and good satisfaction with labor (RR; SE -13.86; 6.40, p=0.033).

**CONCLUSIONS:**

Women from the intervention group had more satisfaction and less pain during labor than women from the non-intervention group.

## INTRODUCTION

Labor and childbirth are major and intense experiences in a woman’s life, associated with joy, expectations, pain, and fear. The World Health Organization (WHO) recommends that all women have the right to experience a positive and safe pregnancy and delivery. Women are entitled to receive respectful, high-quality healthcare, including antenatal education intervention to improve their well-being during childbirth^1^. A good birthing experience is related to the manner in which pregnant women deal with the fear of pain and anxiety and make decisions regarding childbirth. Researchers have described childbirth as a painful phenomenon that may have a positive or negative impact on women’s lives^1^. Childbirth satisfaction^1,2^ and a woman’s psychological and physical well-being^2^ are affected by the expectation of an experience, as well as the lack of autonomy, protagonism and participation in decision-making^2,3^.

Anxiety, fear of childbirth and pain are interrelated and may negatively influence the birthing experience^3^. The high anxiety level may increase epinephrine release and thwart labor progression, increase muscle tone, and hamper the expulsion period^4^. Fear of childbirth, in some settings, can be part of a social construct^5^; it may be due to a negative labor experience^6^ or low self-efficacy^7^, and can be associated with depression and anxiety during pregnancy^8^. Self-efficacy has been described as a cognitive process in which people develop abilities to cope with a certain situation^9^. Therefore, childbirth self-efficacy may be considered an important strategy to control the fear of childbirth and improve satisfaction with birthing. It has been reported that nulliparous women who requested a cesarean delivery referred to a great fear of childbirth^8^; childbirth being a new experience for these women, it is possible that self-efficacy during labor and delivery was low.

Fear of childbirth may have an impact on labor^9^, labor pain and duration, and childbirth^10^ and may be influenced by internal factors like trust, self-efficacy, autonomy, and protagonism in labor^2^. Furthermore, negative feelings decrease when pregnant women can cope with issues related to the birthing experience. Educational activities providing information during antenatal care may help women build confidence and increase their sense of control during labor and satisfaction with childbirth^11^. Antenatal educational activities include non-pharmacological techniques for pain control, increasing childbirth self-efficacy, reducing the fear of childbirth, and decreasing pain during labor and childbirth^10^.

High-risk pregnant women have concerns about the health of their babies and their own health, which may extend to the postpartum period and contribute to increased anxiety^12^, and could be associated with the stress of monitoring pregnancy-related disease and eventual hospital admissions^13^. Also, feelings of guilt, anger, oppression, fear, isolation and apprehension about repeated miscarriages may eventually occur in a high-risk pregnancy, and increase fear of labor and childbirth^14^. For pain control, women may use a breathing technique^15^, an upright position during labor^16,17^ and a relaxation technique^18^. These techniques are safe and may reduce pain, anxiety, and labor duration, and give comfort to the woman^15-18^. However, in some settings, a significant number of pregnant women fail to attend antenatal education^19^, owing to work obligations or house chores^20^.

To the best of our knowledge, there is a paucity of studies on the effectiveness of short educational activities for high-risk pregnant women. Furthermore, there is a lack of information about how to better organize short educational activities that prepare women to be more active in labor control. Our aim was to assess the effectiveness of a short educational intervention based on breathing exercises, relaxation techniques, use of the upright position, and information about labor and birthing offered to high-risk pregnant women during the third trimester of pregnancy (in-person and virtual sessions), and address effectiveness on pain control during labor, postpartum anxiety, and satisfaction with the birthing experience.

## METHODS

### Study design, setting and ethical procedures

We conducted a quasi-experimental study between November 2019 and May 2021 with an intervention group and a control group without intervention at the Women Hospital, University of Campinas, Campinas, SP, Brazil. The study was conducted in a hospital, which is a reference center for teaching, research, and assistance. The protocol was approved on September 30, 2019 by the Ethics Committee of the University of Campinas, Campinas, SP, Brazil (#06769718.0.0000.5404). All participants signed an informed consent form before enrollment in the study. Pregnant women receiving online education intervention were contacted by phone, and the informed consent terms were sent by e-mail.

Antenatal education is offered to all pregnant women in the hospital on days other than routine antenatal consultation days. Pregnant women are assisted during maternity by receiving non-systematic professional guidance on non-pharmacological techniques for pain control during labor. The healthcare professional team that provides this assistance includes physical therapists, nurses, and physicians. In this study, the intervention was based on short antenatal educational activities provided to high-risk pregnant women in the last trimester of pregnancy on the day of antenatal consultation, and an evaluation of intervention efficacy is conducted in the first 24–48 hours postpartum.

### Study participants and sampling

High-risk pregnant women at 36 completed weeks of gestation who attended the outpatient clinic, including women with gestational diabetes, gestational hypertension, risk of preterm birth, infectious diseases during pregnancy and previous clinical conditions such as diabetes, hypertension, and autoimmune diseases, among others, participated in the study. The women were identified a day before antenatal consultation through the hospital’s medical record. Inclusion criteria were women aged 18–35 years, with a singleton fetus, without indication of elective cesarean delivery at the time of study inclusion, and participants with ≥4 hours of labor to have a minimum time to experience labor. Exclusion criteria included women with morbidities like HIV+, cardiac disease, preeclampsia or placental insufficiency, two previous cesarean deliveries, deaf or mute because these women were more likely to have an indication of obstetrics interventions and less than two meetings of educational intervention activities (checked in the postpartum period) because they could compromise the results. The sample size was calculated considering birth satisfaction. The sample size was estimated as 98 participants (49 women per group) to compare birth satisfaction between groups and to compare the proportions between 2 groups for categorical data analysis. The significance level was 5%, and the power of the sample was 80%. One hundred pregnant women were included.

### Procedures

The short antenatal educational intervention was offered on the same day of the antenatal consultation from November 2019 to April 2020. The SARS-CoV-2 (COVID-19) pandemic, with the quarantine and lockdown imposed on the population, interfered with and complicated the implementation of antenatal educational interventions and the participation of pregnant women in these activities, because many health services and providers were obligated to use telemedicine for medical consultations and other health interventions. Due to the restrictions imposed by the COVID-19 pandemic, the antenatal educational intervention was interrupted. To restart educational activities for pregnant women, strategies that could be carried out by telemedicine were implemented. The short antenatal educational activity restarted, with the same selection criteria used initially, changing to a virtual format, maintaining the same content, and continuing to offer the educational activity linked to antenatal consultations. Contact was established with the participant on the day of the antenatal consultation, and the educational activity was scheduled within the next 24 hours after consultation (October 2020 and May 2021). All pregnant women included in the intervention group participated in 2–5 meetings lasting around 20 minutes each.

All educational sessions were similar when performed in-person or virtually (by WhatsApp platform) and began with a brief conversation to encourage women to share their feelings, doubts and concerns about labor and delivery, and continued with information and guidance about the topics based on the needs expressed by the participants. The main topics discussed were contractions, pain during labor and delivery, and how to seek relief by adopting the upright position and using breathing techniques and relaxation. The non-pharmacological techniques were selected because they constitute a complementary approach to pain relief. Once women learn and become confident with their use, they become independent and more active in controlling their birthing process, they can be used by the woman regardless of other resources and are harmless for the baby and the mother.

Breathing techniques, upright positions and relaxation techniques were performed by the pregnant women at all educational meetings. For the breathing technique, women were instructed to take a deep, nasal, slow abdominal inspiration, followed by a deep, prolonged exhalation through the mouth. The women were stimulated to maintain simultaneously global relaxation in the chosen position, mainly an upright position (‘smell the flower and blow out the candle flame at the same time relaxing your body, feel where you are tense, and relax your body’). Among the vertical and comfortable positions during labor, the following positions were trained: sitting on a birthing ball, standing, walking between contractions, squatting, and sitting, and any vertical position that women felt was comfortable and helped to control pain, to perform the breathing techniques and to relax. All the exercises were trained during a time like the duration of a contraction.

All participants from both the intervention and non-intervention groups responded (in-person or by WhatsApp) to a questionnaire about knowledge of non-pharmacological techniques, perception of childbirth, engagement in physical activities and labor pain within 48 hours after delivery. Postpartum women, both those who participated in the short educational intervention and those who participated online before hospital discharge, responded to a questionnaire to assess labor pain and the use of non-pharmacological techniques for pain relief and to answer the STAI questionnaire^21^.

### Outcomes

The main outcomes were labor/childbirth satisfaction, pain during labor and childbirth, and postpartum anxiety. Birth satisfaction was assessed on a 5-point Likert scale in the postpartum period from ‘very bad’, ‘bad’, ‘regular’, ‘good’, to ‘excellent’. The scale was shown to the postpartum patient who selected the option. The ‘very bad’ and ‘bad’ categories were considered to be unsatisfied, and the ‘regular’, ‘good’, and ‘excellent’ categories were considered to be satisfied. To measure labor pain, the pain visual analogue scale (VAS) was used on a scale of 0 to 10, with 0 meaning no pain and 10 being the maximum level of pain tolerated^22^. The participants filled in the VAS scale in the postpartum period within 48 hours after delivery before hospital discharge. Anxiety was measured by the STAI validated in Brazil^21^. The instrument consists of two self-report scales that measure two distinct concepts of anxiety. State anxiety (A-state) is conceptualized as a transient emotional state or condition of the human body that is characterized by feelings of consciously perceived tension and apprehension and by increased activity of the autonomic nervous system. Trait anxiety (A-trait) refers to relatively stable individual differences with a tendency to anxiety, i.e. the difference in tendency to react to situations perceived as threatening with increased intensity of anxiety.

### Data analysis

The Student’s t-test, chi-squared and Fisher’s exact tests were used for analysis. The risk was estimated by the Risk Ratio (RR) with a 95% confidence interval (CI). ANOVA for repeated measures was used to assess the effects on the groups. Categories of anxiety included low and moderate, high and very high. Bivariate linear regression analysis for labor pain included the continuous variables: labor duration (minutes), age (years), gestational age at delivery (weeks), knowledge of non-pharmacological techniques for pain control (yes/no), and categorical variables: type of delivery (vaginal or cesarean), fear of pain (yes/no), anxiety (low, moderate, high, and very high), childbirth satisfaction (very bad, bad, regular, good, excellent), group (intervention/control), previous delivery (yes/no). Multiple linear regression analysis (with stepwise variable selection) was conducted with the variables with p<0.05 to assess the potential relationship between variables and the number of antenatal sessions completed by the intervention group. Variables without normal distribution were transformed into ranks. The SAS System for Windows (Statistical Analysis System), version 9.2 was used.

## RESULTS

One hundred pregnant women were included. The mean age of the women was 27.4 (SD=4.9) years, and regarding physical exercise during pregnancy, 48/50 and 36/50 in the intervention and non-intervention groups, respectively, engaged in some exercise modality (Table 1). The main childbirth experience and neonatal characteristics between women from the intervention and non-intervention groups are shown in Table 2. It can be observed that when we compared women from the intervention to those in the non-intervention group, the latter reported higher fear of pain at labor during antenatal consultations (p<0.013), more women needed analgesia at 0–4 cm of dilation (17/40) (p<0.018), the duration of labor was ≥12 hours (37/50) (p<0.037), while the intervention group reported having a regular, good or excellent experience of labor (36/50) (p=0.014).

**Table 1:** Sociodemographic and obstetric characteristics of women with a high-risk pregnancy in the third trimester of pregnancy of a quasi-experimental study conducted from November 2019 to May 2021 in Brazil (N=100)

*Characteristics*	*Intervention group (N=50) n (%)*	*Non-intervention group (N=50) n (%)*	*p*
**Age** (years)			0.584
≤19	3 (6)	2 (4)	
20–29	32 (64)	27 (54)	
30–35	15 (30)	21 (42)	
**Employment**			0.004^†^
Paid work	37 (74)	22 (44)	
Non-paid work	13 (26)	28 (56)	
**Cohabitation status**			0.117
With a partner	46 (92)	50 (100)	
Without a partner	4 (8)	0	
**Parity**			0.201
Nulliparous	28 (56)	29 (58)	
1 vaginal childbirth	18 (36)	16 (32)	
2 vaginal childbirths	3 (6)	5 (10)	
≥3 vaginal childbirths	1 (2)	0 (0)	
**School education** (years)			0.122
≤8	4 (8)	11 (22)	
9–12	34 (68)	32 (64)	
≥13	12 (24)	7 (14)	
**Practice physical exercises during pregnancy** (yes)	48 (96)	36 (72)	0.129

† Fisher’s exact test.

**Table 2:** Comparison of the main childbirth experience and neonatal characteristics between women from the intervention and non-intervention groups of a quasi-experimental study conducted from November 2019 to May 2021 in Brazil (N=100)

*Variables*	*Intervention (N=50) n*	*Non-intervention (N=50) n*	*p*
**Fear of pain reported during antenatal care** (yes)	29	31	0.683
**Expectations during antenatal care of intense pain during labor** (8 –10 VAS)	50	43	0.013
**Dilatation at admission** (≤3 cm)	33	32	0.325
**Use of analgesia during labor** (yes)	40	37	0.476
**Dilation at the time of analgesia** (cm)*			0.018
0–4	17	7	
5–7	11	12	
8–10	6	15	
**Duration of labor** (hours)			0.037
0–12	13	23	
>12	37	27	
**VAS pain in labor**			
>8	40	44	0.275
**Reported experience of labor**			0.014
Regular, good, excellent	36	24	
Bad, very bad	14	26	
Apgar**			1.000
At 1 min (7–10)	46	46	
At 5 min (7–10)	49	49	

* Without the information of six participants in the intervention group and three participants in the non-intervention group.

** Without information of one participant.

† Fisher’s exact test. (symbol missing from column of p)

Furthermore, the mean VAS for labor pain was 8.8 (SD=0.23) and 9.1 (SD=0.29) among the women from the intervention and non-intervention groups, respectively (p<0.03). After the bivariate analysis for labor pain, we observed a significant relationship between the use of relaxation techniques and reduced labor pain (p=0.024). The comparison between the women from the intervention and non-intervention groups regarding position and use of non-pharmacological techniques during labor is presented in Table 3. Regarding the use of position and non-pharmacological techniques for pain control, women from the intervention group presented higher significant differences in the use of squatting (p<0.032), standing (p<0.047), four supports (p<0.027), and sitting on a birthing ball (p<0.044), when compared to women from the non-intervention group. Furthermore, when compared to the women who attended the educational activities in person or virtually, there were no significant results (data not shown). The multiple regression analysis for labor pain showed a significant relationship between mode of delivery (cesarean delivery: RR; SE -21.43; 5.32, p<0.001) and labor pain, and good satisfaction with labor (RR; SE -13.86; 6.40, p=0.033).

**Table 3:** Comparison between the intervention and non-intervention groups of women with a high-risk pregnancy in the third trimester of pregnancy regarding the position and non-pharmacological techniques used during labor of a quasi-experimental study conducted from November 2019 to May 2021 in Brazil (N=100)

*Position*	*Intervention (N=50) n (%)*	*Non-intervention (N=50) n (%)*	*p*
Squatting	16 (32)	7 (14)	0.032*
Standing	44 (88)	35 (70)	0.047*
Lying down	40 (80)	41 (82)	1.000
Sitting	38 (76)	38 (76)	1.000
Four supports	10 (20)	2 (4)	0.027†
Sitting on a ball	32 (64)	22 (44)	0.044†
Kneeling	7 (14)	2 (4)	0.159
Massage	33 (66)	34 (68)	1.000
Use of shower	38 (76)	39 (78)	1.000
Use of breathing	49 (98)	45 (90)	1.000
Relaxation	38 (76)	29 (58)	0.313
De-ambulation	42 (84)	33 (66)	0.476

* Chi-squared test.

† Fisher’s exact test.

## DISCUSSION

The results of this study show that women participating in short antenatal education in person or online (intervention group), reported having a regular, good, or excellent experience of labor, while women in the non-intervention group reported higher fear of pain at labor during antenatal consultations, more women needed analgesia at 0–4 cm of dilation, and the duration of labor was ≥12 hours. In addition, there was a significant relationship between the use of relaxation techniques and the position adopted during labor and reduced labor pain. Regarding the use of non-pharmacological techniques for pain control, women from the intervention group presented higher significant differences in the use of squatting, standing, four supports, and sitting on a birthing ball when compared to women from the non-intervention group. However, when compared to the women who attended the educational activities in person or virtually, there were no significant results.

According to the women’s perception from both groups, pain was intense. The intervention group reported higher birth satisfaction and less unsatisfying labor experience than the non-intervention group. Concerning labor pain, women from the intervention group, either from the in-person or online group, had more autonomy, less fear, knew breathing techniques, sought upright positions, used relaxation techniques, and were more confident about labor pain control.

Our results agree with previous reports showing that antenatal education may have a positive impact on fear reduction and increased self-efficacy of pregnant women during labor and childbirth^10,23^. Pregnant women reported feeling more self-assured when they were prepared for the emotional and physical challenges of labor because they knew what to expect and were familiar with the childbirth process^2^. However, controversy still exists in the literature concerning educational interventions. Another study reported that pregnant women participating in antenatal education did not reflect the number of women who reported less pain during labor^24^. On the other hand, in a study with high-risk pregnant women, the authors found that women reported decreased labor pain, and that the healthcare providers involved in the management of high-risk pregnant women were vigilant and present during antenatal care to support and prevent potential complications^25^.

Pregnant women from the intervention group had higher scores in pain control. This was due to their greater autonomy and knowledge of the importance of remaining in different upright positions. Recent studies showed that the kneeling squat position^26^ and squatting position^27^ are optimal for increasing pelvic outlet capacity. This enables the sacrum and coccyx to have more freedom of movement and facilitates the passage of the fetus into the maternal pelvis^27^. Another benefit of the upright position is that it allows gravity to act more efficiently on fetal descent^28^.

We found that childbirth satisfaction was higher in the intervention group. Evidence suggests that the protagonism of the pregnant woman in the decision-making process in labor and childbirth is a powerful influence on women’s childbirth satisfaction. Feeling left out from decision-making may cause a negative and traumatic experience^29^. Although there were no significant differences in perceived pain during labor between the intervention and non-intervention groups, higher childbirth satisfaction in the intervention group was possibly related to the three non-pharmacological techniques used for labor pain control practised during the intervention. It should be highlighted that helping women in labor to have a satisfactory birthing experience should be a priority in maternity health services, according to current recommendations for humanized childbirth.

The WHO emphasizes that pregnant women should be allowed to make decisions about labor and pain control during labor. Our results showed that pregnant women from the intervention group chose more birthing positions than women from the non-intervention group. Decision-making is fundamental for the sense of control. For most women, involvement in decision-making had a positive impact on the labor experience. It is well documented that high-risk pregnant women feel less confident to actively participate in clinical decision-making for fear of putting their baby and themselves at risk^30^. So, it is legitimate to suppose that this may have been one of the reasons for not having more differences in the results between groups regarding the active use of non-pharmacological techniques for pain control during labor.

It was evident in our study that all participants had the support of a companion of their choice during labor and received excellent care during antenatal care in a specialized service for high-risk pregnancies. Irrespective of the support received, women participating in the short antenatal educational intervention developed a higher sense of security and satisfaction. Women in the intervention group also reported more pain relief than women in the non-intervention group. It is reasonable to assume that these outcomes may have resulted from knowledge of non-pharmacological techniques and increased autonomy during labor and childbirth. These factors are associated with feelings of satisfaction, security and respect, as well as increased self-efficacy and body control during labor^31^.

### Limitations

A possible limitation of our study was that the intervention of the study group required two modalities (in-person and online) due to the restrictions imposed by the COVID-19 pandemic. However, online meetings, such as obstetric consultations, were shown to be fundamental during social isolation and offered several advantages. According to some studies, these meetings were well-accepted by pregnant women^32^ and viable for healthcare professionals^33^. On the other hand, the strength of our study was that the same intervention model was used to organize the online intervention. The use of the same intervention was shown to be efficient. In addition, the antenatal educator did not find it difficult to work online, nor were there complaints from the participants (observational and oral reports).

## CONCLUSIONS

Our study showed that a short antenatal educational intervention (in person or online) offered during the last trimester of pregnancy at the time of antenatal consultation, in high-risk obstetric service, benefits both nulliparous and parous women, increases both pain control during labor and satisfaction with labor and childbirth. Further studies with a larger sample size are needed to confirm the results of the current study.

## Data Availability

The data supporting this research are available from the authors upon reasonable request.
